# Impact of post-alignment processing in variant discovery from whole exome data

**DOI:** 10.1186/s12859-016-1279-z

**Published:** 2016-10-03

**Authors:** Shulan Tian, Huihuang Yan, Michael Kalmbach, Susan L. Slager

**Affiliations:** 1 Division of Biomedical Statistics and Informatics, Department of Health Sciences Research, Mayo Clinic, 200 1st St SW, Rochester, MN 55905 USA; 2 Division of Research and Education Support Systems, Department of Information Technology Mayo Clinic, Rochester, MN 55905 USA

**Keywords:** Base quality score recalibration, Human leukocyte antigen, Local realignment, Variant calling, Whole exome sequencing

## Abstract

**Background:**

GATK Best Practices workflows are widely used in large-scale sequencing projects and recommend post-alignment processing before variant calling. Two key post-processing steps include the computationally intensive local realignment around known INDELs and base quality score recalibration (BQSR). Both have been shown to reduce erroneous calls; however, the findings are mainly supported by the analytical pipeline that incorporates BWA and GATK UnifiedGenotyper. It is not known whether there is any benefit of post-processing and to what extent the benefit might be for pipelines implementing other methods, especially given that both mappers and callers are typically updated. Moreover, because sequencing platforms are upgraded regularly and the new platforms provide better estimations of read quality scores, the need for post-processing is also unknown. Finally, some regions in the human genome show high sequence divergence from the reference genome; it is unclear whether there is benefit from post-processing in these regions.

**Results:**

We used both simulated and NA12878 exome data to comprehensively assess the impact of post-processing for five or six popular mappers together with five callers. Focusing on chromosome 6p21.3, which is a region of high sequence divergence harboring the human leukocyte antigen (HLA) system, we found that local realignment had little or no impact on SNP calling, but increased sensitivity was observed in INDEL calling for the Stampy + GATK UnifiedGenotyper pipeline. No or only a modest effect of local realignment was detected on the three haplotype-based callers and no evidence of effect on Novoalign. BQSR had virtually negligible effect on INDEL calling and generally reduced sensitivity for SNP calling that depended on caller, coverage and level of divergence. Specifically, for SAMtools and FreeBayes calling in the regions with low divergence, BQSR reduced the SNP calling sensitivity but improved the precision when the coverage is insufficient. However, in regions of high divergence (e.g., the HLA region), BQSR reduced the sensitivity of both callers with little gain in precision rate. For the other three callers, BQSR reduced the sensitivity without increasing the precision rate regardless of coverage and divergence level.

**Conclusions:**

We demonstrated that the gain from post-processing is not universal; rather, it depends on mapper and caller combination, and the benefit is influenced further by sequencing depth and divergence level. Our analysis highlights the importance of considering these key factors in deciding to apply the computationally intensive post-processing to Illumina exome data.

**Electronic supplementary material:**

The online version of this article (doi:10.1186/s12859-016-1279-z) contains supplementary material, which is available to authorized users.

## Background

Genetic variation is associated with the etiology of human disease and drug response [1–3]. Completely cataloguing the variants in individual genomes, which has been pursued largely through whole genome or whole exome sequencing effort, is essential in disease diagnosis and pharmacogenomics studies [4, 5]. Whole exome sequencing is widely used in clinical settings, owing to the lower cost compared to whole genome sequencing and the remarkable success in identifying causative mutations underlying Mendelian diseases [6–8].

Variants (SNPs and short INDELs) in sequencing data are identified mainly through mapping-based approaches [9–11], in which raw sequencing reads are first mapped to a reference sequence and the sites differing between reads and the reference are then identified by variant calling [9, 12]. Several variant calling algorithms have been developed, such as SAMtools [13], the Genome Analysis Toolkit (GATK) UnifiedGenotyper and HaplotypeCaller [14], FreeBayes [15], Platypus [8], Atlas2 Suite [16], and the SNP and INDEL callers in the Short Oligonucleotide Analysis Package (SOAP, http://soap.genomics.org.cn/) [17]. Of these, FreeBayes, GATK HaplotypeCaller and Platypus are haplotype-based callers which implement De Bruijn graph-based local assembly [8, 14] or construct haplotypes directly from mapped reads [15].

As outlined in the GATK Best Practices [18], some variant discovery pipelines perform post-processing of alignments prior to variant calling, with the expectation that this practice would improve variant calling accuracy [19]. The post-processing typically includes duplicate marking, local realignment around known INDELs, and base quality score recalibration (BQSR) [4].

Duplicates are pairs of reads that are mapped to the same genomic location and the same strand. They are prevalent in both whole genome and exome sequencing data [12] and are believed to be artifacts from the polymerase chain reaction (PCR) amplification of the same DNA molecule during library preparation [4]. The inclusion of duplicates more likely gives rise to erroneous calls because the presence of duplicates would alter the ratio of reads supporting one allele versus the other at heterozygous sites; duplicate reads might also carry errors introduced by the PCR amplification, thus further complicating the variant calling [17]. Duplicates can be identified using Picard command-line tool MarkDuplicates (https://broadgithub.io/picard/command-line-overview.html), such that only one of the duplicates will be used in the subsequent variant calling. Marking duplication is more effective for INDEL calling than for SNP calling [12], and in cases when the coverage is low [20].

Reads spanning INDELs have a high chance of being aligned incorrectly to the reference [20, 21]. A previous study revealed that BWA mapping generated misalignment for over 15 % of the reads spanning known homozygous INDELs [4]. Without correction, those misaligned bases in reads can be easily called as spurious variants.

Finally, the confidence level in variant calling depends on the accuracy of both base calling and base quality score [22]. The latter measures the probability that a base is called incorrectly [23], with a Phred-scale quality score of Q ideally corresponding to an error rate of 10^- Q/10^ [22]. However, the raw quality score does not truly reflect the base-calling error rate; instead, it varies with multiple factors including sequencing platform, the number of machine cycles at which a base is sequenced, and the sequence composition [4, 22]. For example, the bases at the 3′ end of a read are typically more error prone than those at the 5′ end [17]. The GATK BaseRecalibrator and PrintReads commands can be used to recalibrate the quality score, thereby to improve variant calling accuracy [22].

The effect of local realignment and BQSR has been assessed using both whole genome and whole exome sequencing data, mostly for BWA together with GATK UnifiedGenotyper, GATK HaplotypeCaller and SAMtools [2, 4, 12, 20]. However, the results are inconsistent. The earliest study revealed that local realignment corrected misalignments at approximately 1.8 million sites in the whole genome data and over 100,000 sites in the whole exome data from NA12878 [4]. They found that duplicate marking, local realignment and BQSR together removed about 2.5–6.0 % of the raw SNP calls, of which the vast majority were false positives. Using exome data from breast cancer patients, a similar effect was detected for local realignment but not for BQSR in GATK UnifiedGenotyper and SAMtools calling [20]. Furthermore, using high (55-65x) coverage whole genome sequencing data in NA12878, Li [12] found no effect from both local realignment and BQSR for the above three callers. As local realignment and BQSR were evaluated only for a few variant discovery methods, their effectiveness for other mappers and callers remains unexplored. Local realignment is a computationally intensive process. In principle, haplotype-based callers apply local de novo assembly (like GATK HaplotypeCaller and Platypus) or build haplotype directly from mapped reads (like FreeBayes), raising the question whether local realignment is indeed needed for these callers.

Finally, in BQSR, the algorithm needs to identify a list of supposedly non-polymorphic sites that do not overlap with any known polymorphic sites (like those in dbSNP) and that do not match the reference sequence either. It then builds a linear model taking into account the raw base quality score, base position in the reads (i.e., sequencing cycle), as well as the dinucleotide composition of those non-polymorphic sites, by which the recalibrated quality score is computed [4, 22]. While it is relatively straightforward to identify non-polymorphic sites in ordinary genomic regions where sequence diverges at the level of only about 0.1 % [24], difficulty arises in regions of high divergence. In the latter case, a portion of the non-polymorphic sites likely represents true variants. In fact, there are over one hundred highly divergent regions in the human genome [25], and some reach the level of 10–15 % divergence between haplotypes [26]. Many are clinically important, including the well-known human leukocyte antigen (HLA) region on chromosome 6p21 that is implicated in more than 100 diseases [27–29]. Thus, the effect of BQSR on variant calling in such highly divergent regions needs to be assessed separately.

In this study, we sought to examine the impact of local realignment and BQSR across a panel of 25 variant discovery methods on simulated data and 30 methods on real exome data, taking into account both coverage depth and divergence level. We began with simulated data and extended to five public exome-seq data in NA12878, which were generated using two exome capture kits on three Illumina HiSeq platforms. We found that local realignment mainly impacts INDEL calling and BQSR largely affects SNP calling. In INDEL calling, noticeable effect of local realignment was restricted to only a few methods, with no effect on INDEL detection from Novoalign alignment and minor effects on the three haplotype-based callers. In the latter, BQSR reduces SNP calling sensitivity for many of the methods. Thus, consideration should be given to both mapper and caller when applying post-processing to Illumina exome data.

## Methods

### Simulation of exome-seq reads

Simulation of reads provides an ideal approach for initial assessment of individual methods in a controlled situation where the “true” variants are predefined [30]. We focused on chromosome 6p21.3, which contains the highly divergent HLA region. To simulate exome-seq reads from this chromosome, we generated candidate regions by merging hg19 refGene exons (as of 12/10/2013, extended by +/-100 bp) with regions interrogated by any of the four Agilent SureSelectXT Human All Exon kits (All Exon 50Mbp, All Exon V4, All Exon V4 + UTRs, and All Exon V5 + UTRs, http://www.agilent.com).

Simulation was done using Dwgsim v0.1.11 (https://github.com/nh13/DWGSIM/wiki) at six mutation rates (0.05, 0.01, 0.5, 1, 5 and 10 %). Illumina paired 100-base reads were simulated to an average per-base coverage of 100x, which were down-sampled to 40 and 5x coverage. Dwgsim was run with the following parameter settings: SNP-to-INDEL ratio of 9:1 per mutation rate, outer distance of 200 bp between paired reads, no error, no random DNA and a random seed of 123. Instead of providing a list of candidate mutations (parameter -v), we generated random mutations by specifying a mutation rate (parameter -r) and the fraction of INDELs over the total mutations (parameter -R), with an equal probability of insertions and deletions and a geometric distribution of INDEL sizes. To enable the assessment of BQSR, rather than relying on the dummy base quality scores assigned by Dwgsim, we instead gave each read a string of empirical base quality scores randomly taken from a pool of real Illumina sequencing data. These came from our internal 100-bp exome-seq data in chronic lymphocytic leukemia patients. A similar approach was adopted by the sequence simulation tool ART, which uses base quality scores from real sequencing data [31].

### Mapping and post-processing of simulated reads

Simulated reads were aligned to the hg19 human reference sequence using five mappers, including BWA-backtrack (referred to as BWA) [13], GSNAP [32], NextGenMap [33], Novoalign (http://www.novocraft.com/), and Stampy [24]. Unlike BWA, the other four mappers could map reads to highly divergent regions. The parameter settings for each mapper were provided in Additional file 1. Alignments in the sequence alignment/map (SAM) format were converted into the binary alignment/map (BAM) format using SAMtools [34], and sorted by coordinate using the SortSam command in the Picard tools (http://broadinstitute.github.io/picard/). Alignments from all chromosomes were processed sequentially through duplicate marking, local realignment and BQSR.

Duplicates were marked using the Picard MarkDuplicates command. We then performed local realignments around 90 % of all simulated INDELs using GATK IndelRealigner command. We chose 90 % by considering the fact that, in real exome data, only known rather than all INDELs are used for local realignment. Subsequently, the GATK BaseRecalibrator and PrintReads commands were used in BQSR using 90 % of simulated variants. To investigate how these two post-processing steps impact variant calling, variants were identified both before and after each step, as below.

### Variant calling from simulated data

We selected five popular variant callers, including GATK UnifiedGenotyper and HaplotypeCaller [4, 14], FreeBayes [15], SAMtools mpileup with BCFtools consensus-caller (referred to as SAMtools) [34], and Platypus [8]. The parameter settings for each of the callers were provided in Additional file 1. To increase the comparability among these callers, the multiple-nucleotide polymorphisms (i.e., multiple SNPs within five bases that are reported as a single event) reported by Platypus and FreeBayes were decomposed into individual variants using GATK walker VariantsToAllelicPrimitives. Considering that the boundaries of INDELs are difficult to define precisely, overlap was inferred if an INDEL was called within five bases of a permuted INDEL.

Variant calling sensitivity and precision rate were estimated using GATK walker GenotypeConcordance, where sensitivity is estimated as “true positive/(true positive + false negative)” and precision rate as “true positive/(true positive + false positive)”. Here, true positive refers to the number of true (simulated) variants identified by a method, false negative represents the number of true variants missed by a method, and false positive represents the number of called variants that do not overlap the true variants. The effect of local realignment was measured as the change in sensitivity and precision rate before and after local realignment; BQSR effect was measured similarly.

### Variant calling from exome-seq data in NA12878

We further assessed local realignment and BQSR using exome-seq data from NA12878, in which several call sets have been generated from whole genome and exome sequencing data. We first compiled a list of “true” variants (referred to as public call set) by combining the two variant lists below. Those variants were identified through whole genome and whole exome sequencing, involving seven sequencing platforms, seven mappers and four callers. A high-confidence call set was generated through integrative analyses of 11 whole genome and 3 exome sequencing data (ftp://ftp-trace.ncbi.nih.gov/giab/ftp/data/NA12878/analysis/GIAB_integration/NIST_RTG_PlatGen_merged_highconfidence_v0.2.primitives.vcf.gz) [35]. To minimize bias toward a specific analytical pipeline, seven mappers together with three callers, Cortex [36], GATK UnifiedGenotyper and GATK HaplotypeCaller, were used. The second list represents a union of three call sets, which were generated using Cortex, DISCOVAR and GATK HaplotypeCaller from a PCR-free genomic library sequenced to 250 base pairs (ftp://ftp.broadinstitute.org/pub/crd/DiscovarManuscript/vcf/) [5]. Although we also used GATK UnifiedGenotyper and HaplotypeCaller, the bias toward these two callers should be minimal because the public call set also used other callers and we assessed the effect of post-alignment processing based on the change of sensitivity rather than the sensitivity itself.

We downloaded five paired-end exome-seq data in NA12878 (66-100x coverage; Additional file 2: Table S1). The datasets were chosen to represent two different capture kits and three Illumina sequencing platforms. The Roche SeqCap EZ Human Exome kit has 64 megabases of capture regions, versus only 37 megabases interrogated by the Illumina Nextera Rapid Capture Exome kit. For the two 150-bp datasets, only the first 100 bases were used. To understand how the effect of post-processing varies across different coverage depths, each full (66-100x) dataset was down-sampled to 40, 20 and 10x coverage using SAMtools.

Reads mapping, post-processing and variant calling followed the procedure used in the simulated data, with two modifications in local realignment and BQSR. First, local realignment was performed around the Mills and 1000G gold standard INDELs (Mills_and_1000G_gold_standard.indels.hg19.vcf.gz). Second, BQSR was conducted using known variants in dbSNP v135 (downloaded as part of the GATK bundle) together with the Mills and 1000G gold standard INDELs. In addition to the 5 callers and 5 mappers used for simulated data, we also assessed the effects of local realignment and BQSR for the BWA-MEM algorithm [37] and SAMtools/BCFtools multiallelic-caller (parameter settings in Additional file 1). Released in February 2013, BWA-MEM was developed mainly for aligning sequences from 70 bp up to 1 Mbp. It outperforms the BWA-backtrack algorithm in mapping long (> = 70-bp) reads [37]. The earlier releases of SAMtools/BCFtools (v0.1.19 or older, with consensus-caller) assume biallelic sites without handling multiallelic variants properly. They only take the strongest non-reference allele, which may not be optimal for more complex genomes, such as a cancer genome (http://samtools.sourceforge.net/mpileup.shtml). The multiallelic calling model is recommended in the later release.

The GRCh38 (hg38) reference assembly presents a better representation of the human genome by filling many gaps and including 261 alternate loci across 178 regions (http://www.ncbi.nlm.nih.gov/projects/genome/assembly/grc/human/). However, many of the mapping tools are not capable of handling the alternate loci; thus, the inclusion of alternate loci in the reference would cause more reads to have an “ambiguous” mapping status. In this study, we attempted to assess the effects of local realignment and BQSR on hg38. The hg38 full assembly together with the hs38d1 decoy, the HLA sequences and ALTs were downloaded from ftp://ftp.1000genomes.ebi.ac.uk/vol1/ftp/technical/reference/GRCh38_reference_genome/. We mapped reads from NA12878_01 and NA12878_04 (approximately 100x coverage; Additional file 2: Table S1) to the hg38 reference using the ALT-aware aligner BWA-MEM (v0.7.12). BWA-MEM alignments were first processed using the script “bwa-postalt.js” available in the bwakit (v0.7.12) software package (https://github.com/lh3/bwa/tree/master/bwakit). This script re-assigns mapping quality scores for reads with hits in the ALT contigs. Duplicates were marked by the Picard MarkDuplicates command. Local realignment and BQSR were performed using the commands (IndelRealigner, BaseRecalibrator and PrintReads) available in GATK v3.3-0. The known INDELs used for local realignment were combined from two sources: ftp://ftp.1000genomes.ebi.ac.uk/vol1/ftp/technical/reference/GRCh38_reference_genome/other_mapping_resources/ALL.wgs.1000G_phase3.GRCh38.ncbi_remapper.20150424.shapeit2_indels.vcf.gz and ftp://ftp.1000genomes.ebi.ac.uk/vol1/ftp/technical/reference/GRCh38_reference_genome/other_mapping_resources/Mills_and_1000G_gold_standard.indels.b38.primary_assembly.vcf.gz. The list of known SNPs used for BQSR was downloaded from ftp://ftp.1000genomes.ebi.ac.uk/vol1/ftp/technical/reference/GRCh38_reference_genome/other_mapping_resources/ALL_20141222.dbSNP142_human_GRCh38.snps.vcf.gz.

While post-processing was performed genome-wide across all chromosomes, variants were identified only from chromosome 6. Variants with a phred-scale quality of at least 20 were retained, which were separated into “known” and “novel” ones by intersecting with dbSNP v138 (ftp://gsapubftp-anonymous@ftp.broadinstitute.org/bundle/2.8/hg19/dbsnp_138.hg19.vcf.gz, for hg19) or dbSNP v142 (for hg38). The public call set was also split into “known” and “novel”. Hg19 to hg38 liftover was performed using CrossMap [38]. By treating the known SNPs in the above public call set as “true” positives, the sensitivity for known SNPs was estimated as “true positive/(true positive + false negative)”. The true positive refers to the number of known SNPs identified by a method that are in the public call set; false negative represents the number of known SNPs missed by a method but are in the public call set. The sensitivity for known INDELs was estimated similarly.

## Results and discussion

We used both simulated reads from exonic regions of chromosome 6 and five exome data in NA12878. We evaluated, for each of the variant discovery methods, how local realignment and BQSR might impact the outcome across different divergence levels and coverage depths.

### Impact of local realignment in simulated data

**Fig. 1:**
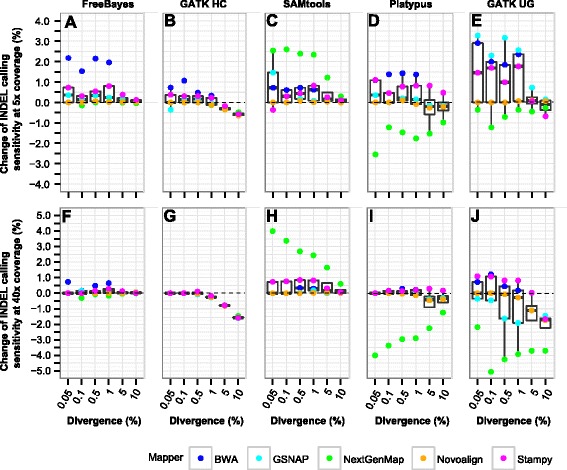
Box plot showing the change of INDEL calling sensitivity after local realignment. **a**–**e** Simulated datasets with 5x coverage. **f**–**j** Simulated datasets with 40x coverage. Local realignment was performed using 90 % of the preplaced INDELs. The change of INDEL calling sensitivity is calculated as the sensitivity after local realignment, subtracted by that after duplicate marking. For callers together with BWA, only datasets with 0.05–1 % divergence were used. GATK HC, GATK HaplotypeCaller; GATK UG, GATK UnifiedGenotyper

We assessed a total of 280 cases from five mappers, five callers, two coverage depths (5 and 40x), and six divergence levels. BWA was excluded at 5–10 % divergence. Local realignment had nearly no impact on SNP calling sensitivity (Additional file 2: Table S2) and increased precision rate slightly (0.2–0.3 %) in 11 cases involving GATK UnifiedGenotyper and Platypus (Additional file 2: Table S2). Next, we sought to understand to what extent local realignment might impact INDEL calling. We observed a 0.4–1 % increase of precision rate in 16 (5.7 %) cases in INDEL calling (Table 1). These cases used SAMtools or GATK UnifiedGenotyper calling from Stampy alignment (Additional file 1: Figure S1). Little or no effect was detected in the other cases, including all from the three haplotype-based callers (Table 1; Additional file 1: Figure S1). On the other hand, local realignment led to an obvious gain or loss of sensitivity (by 1–5 %) in approximately one-fifth of the cases (Table 1). The change of INDEL calling sensitivity depended on multiple factors (Fig. 1), which we described in detail below.

**Table 1:** Effect of local realignment in INDEL calling

Metrics	Change (%)	Cases	
		No.	%
Sensitivity	−5.1 – −4	2	0.71
Sensitivity	−4 – −3	5	1.79
Sensitivity	−3 – −2	5	1.79
Sensitivity	−2 – −1	17	6.07
Sensitivity	−1 – −0.2	34	12.14
Sensitivity	−0.2–0.2	129	46.07
Sensitivity	0.2–1	53	18.93
Sensitivity	1–2	19	6.79
Sensitivity	2–3	12	4.29
Sensitivity	3–4	4	1.43
Precision rate	−0.2–0	33	11.79
Precision rate	0–0.2	219	78.21
Precision rate	0.2–0.4	12	4.29
Precision rate	0.4–0.6	8	2.86
Precision rate	0.6–0.8	5	1.79
Precision rate	0.8–1	3	1.07

Alignments were subjected to duplicate marking and then to local realignment. A total of 280 cases were evaluated, which represent combinations among five mappers, five callers, six divergence levels and two coverage depths (5 and 40x), excluding 20 cases with BWA mapping at 5–10 %. In each case, the change of INDEL calling sensitivity is calculated as the sensitivity after local realignment using 90 % simulated INDELs, subtracted by that after duplicate marking. The change in precision rate is calculated in the same way

In INDEL calling, the impact of local realignment on sensitivity is mapper-, caller- and coverage-dependent. As for the mappers, NextGenMap was affected the most and Novoalign the least (Fig. 1). For Novoalign, nearly no changes in sensitivity were detected at low divergence (Fig. 1), likely reflecting the optimal alignments achieved by the underlying full Needleman-Wunsch algorithm. On the other hand, local realignment increased the sensitivity of NextGenMap with SAMtools (Fig. 1c and h) but decreased its sensitivity with Platypus (Fig. 1d and i) and GATK UnifiedGenotyper (Fig. 1e and j). A previous study showed that local realignment improved GATK UnifiedGenotyper calling accuracy on BWA alignment [4]. We indeed found that it generally increased sensitivity for BWA with all the callers at low (0.05–1 %) divergence, more obvious at 5x coverage (Fig. 1a-e). For GSNAP, local realignment increased the sensitivity at 5x but decreased the sensitivity at 40x coverage in GATK UnifiedGenotyper calling (Fig. 1e and j). Of the callers, overall GATK HaplotypeCaller, Platypus and FreeBayes were less affected at 40x coverage and low divergence, compared to the other two callers (Fig. 1f–j). A similar finding was reported for GATK HaplotypeCaller in exome-seq data [39]. These haplotype-based callers are capable of alleviating alignment ambiguity around INDELs internally through local de novo assembly [8, 18] or direct construction of haplotypes from alignments [15]. For these callers, the benefit of applying local realignment in GATK Best Practices would be minimized.

The role of local realignment in INDEL calling also depends on divergence. At 0.05–1 % divergence, 33 cases showed increase of sensitivity (> = 1 %) after local realignment, versus only 2 cases (NextGenMap + SAMtools) at 5–10 % divergence. Whereas, of the 29 cases whose sensitivity decreased by > =1 %, about half were from 5–10 % divergence. In summary, local realignment mainly affects INDEL calling sensitivity, more conspicuous at low coverage. When the coverage and divergence are both low, local realignment tends to increase the sensitivity. At high coverage and low divergence, local realignment has nearly no impact on the haplotype-based callers. Of the five mappers, Novoalign is least affected, in contrast with BWA that generally shows an increase of sensitivity.

### Impact of BQSR in SNP calling of simulated data

We evaluated the effect of BQSR by measuring the change in sensitivity and precision rate between BQSR and local realignment. To ensure meaningful comparison, we limited the analysis to a subset of the cases. In assessing the change in sensitivity, we required that the sensitivity prior to BQSR (after local realignment) should exceed 35 %, while in assessing the change in precision rate, we further required that the sensitivity should not be decreased by more than 15 % after BQSR.

**Fig. 2:**
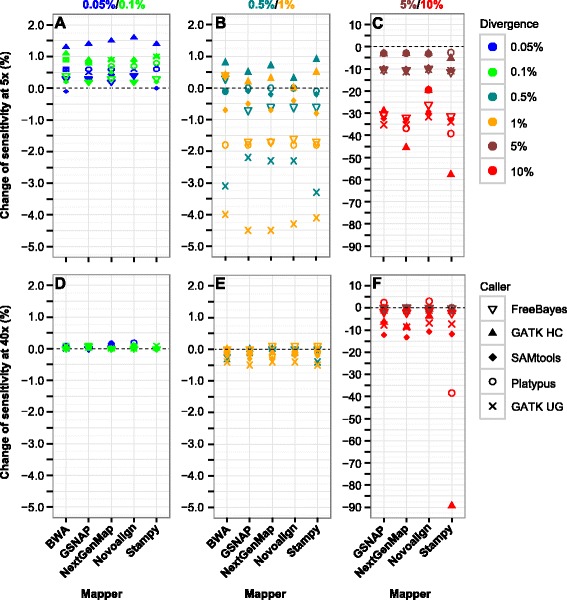
Change of SNP calling sensitivity after BQSR. **a**–**c** Datasets with 0.05–0.1, 0.5–1 and 5–10 % divergence, respectively, at 5x coverage. **d**–**f** Datasets with 0.05–0.1, 0.5–1 and 5–10 % divergence, respectively, at 40x coverage. The change of sensitivity is calculated as the sensitivity after BQSR using 90 % of the permuted SNPs, subtracted by that after local realignment using 90 % of the preplaced INDELs. For callers together with BWA, only datasets with 0.05–1 % divergence were used. GATK HC, GATK HaplotypeCaller; GATK UG, GATK UnifiedGenotyper

The impact of BQSR on SNP calling is striking at low coverage and high divergence (Fig. 2). We observed a trend of decrease in sensitivity by BQSR following the increase in divergence. However, at low divergence and 40x coverage, of the five callers, only GATK UnifiedGenotyper showed a small (0.3–0.5 %) decrease in sensitivity at 0.5–1 % divergence (Fig. 2d and e; Table 2). Nevertheless, at 5x coverage the effect was more obvious for several callers (Fig. 2a and b; Table 2). More specifically, BQSR largely increased the sensitivity at 0.05–0.1 % divergence; at 0.5–1 % divergence, it increased the sensitivity for GATK HaplotypeCaller but decreased the sensitivity for GATK UnifiedGenotyper and Platypus. At 5–10 % divergence, BQSR reduced the sensitivity in vast majority of the cases (Fig. 2c and f; Table 2); at 5x coverage, sensitivity was decreased by 15.5 % (median), versus 1.5 % at 40x coverage (Table 2).

**Table 2:** Effect of BQSR in SNP and INDEL calling

Type	Metric	Div	5x coverage				40x coverage			
		(%)	Case	Min	Max	Mdn	Case	Min	Max	Mdn
SNP	Sensitivity	0.05–0.1	50	−0.1	1.6	0.6	50	0	0.2	0
SNP	Sensitivity	0.5–1	50	−4.5	0.9	−0.6	50	−0.5	0.1	−0.1
SNP	Sensitivity	5	20	−11.4	−2.6	−9.8	20	−1.8	0.2	−0.5
SNP	Sensitivity	10	20	−57.8	−19.5	−32.3	20	−89.4	3	−7.1
SNP	Precision rate	0.05–1	100	−0.1	0.3	0	100	−0.2	0.1	0
SNP	Precision rate	5	20	0	0.3	0.1	20	0	0.2	0
SNP	Precision rate	10	0	-	-	-	18	−0.3	1.5	0.2
INDEL	Sensitivity	0.05–0.1	40	−1.5	1.8	0.4	50	−0.2	0	0
INDEL	Sensitivity	0.5–1	40	−0.3	1	0.1	50	−0.1	0.1	0
INDEL	Sensitivity	5	12	−6.4	−0.1	−1.4	20	−0.9	2.8	0
INDEL	Sensitivity	10	12	−51.9	−2.4	−34.6	16	−75.3	0.1	−9.7
INDEL	Precision rate	0.05–1	80	−0.1	0	0	100	−0.1	0.2	0
INDEL	Precision rate	5	12	0	0.3	0	20	0	0.8	0
INDEL	Precision rate	10	0	-	-	-	13	−0.2	3.2	0

Between 5 and 25 mapper-caller combinations were assessed at each of the six divergence levels and two coverage depths in simulation, excluding combinations with BWA at 5–10 % divergence. They were selected to have a sensitivity of at least 35 %; to estimate the change in precision rate, we also required that sensitivity should not decrease by more than 15 % after BQSR. *Div* divergence, *Mdn* median

There were only a few cases where BQSR altered the precision rate. Overall the effect was negligible at 0.05–5 % divergence (Table 2). At 10 % divergence, we excluded 22 cases whose sensitivity was decreased by over 20 % after BQSR. In the remaining 18 cases, all at 40x coverage, only SAMtools and GATK UnifiedGenotyper showed a 0.2–0.5 % increase and Platypus (with GSNAP and NextGenMap) showed an 1–1.5 % increase in precision rate.

### Impact of BQSR in INDEL calling of simulated data

**Fig. 3:**
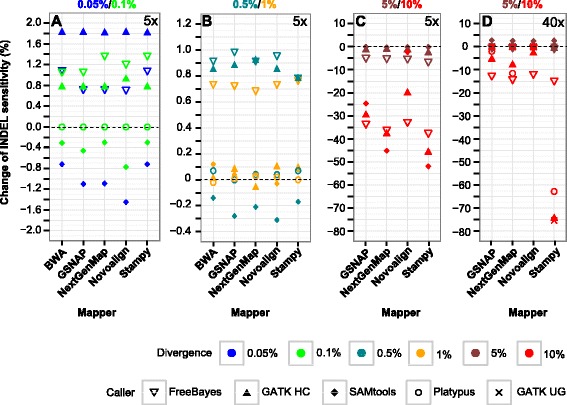
Change of INDEL calling sensitivity after BQSR. **a**–**c** Datasets with 0.05–0.1, 0.5–1 and 5–10 % divergence, respectively, at 5x coverage. **d** Datasets with 5–10 % divergence and 40x coverage. Datasets with 0.05–1 % divergence and 40x coverage were not displayed, since there was nearly no change in sensitivity after BQSR. See Fig. 2 legend for more information

Compared to the three haplotype-based callers, GATK UnifiedGenotyper and SAMtools are less sensitive in INDEL calling (Tian et al., unpublished results). In assessing the impact of BQSR on sensitivity, we excluded 40 cases from the two callers due to their low (<35 %) sensitivity, mainly at 5x coverage. In two-thirds of the remainder, including all those at 0.05–1 % divergence and 40x coverage, BQSR had little effect on sensitivity (<=0.3 % change, Table 2). In the other one-third, BQSR either increased (39 cases, by 0.7–2.8 %) or decreased (39 cases, by 0.5–75.3 %) sensitivity (Table 2). The former was predominantly from 5x coverage and 0.05–1 % divergence (Fig. 3a and b) and the latter from high divergence (Fig. 3c and d), which we discussed below.

For GATK UnifiedGenotyper, the analysis was limited to the 40x coverage (see Methods). For GATK UnifiedGenotyper (with Stampy only) and Platypus, BQSR decreased the sensitivity only at 10 % divergence (Fig. 3c and d). FreeBayes and GATK HaplotypeCaller were similarly impacted; BQSR increased the sensitivity at 5x coverage and < =1 % divergence (Fig. 3a and b), but decreased the sensitivity at 5x coverage and 5–10 % divergence (Fig. 3c) and at 40x coverage and 10 % divergence (Fig. 3d). Finally, for SAMtools, however, BQSR increased the sensitivity at 40x coverage and 5 % divergence (Fig. 3d) but generally reduced the sensitivity at 5x coverage and 0.05–0.1 % divergence (Fig. 3a). On the other hand, 95 % of the cases showed nearly no (<0.1 %) change in precision rate (Table 2).

### Impact of local realignment in NA12878 exome data

Given the limitations in simulated data [12], we further assessed local realignment and BQSR over five exome-seq data in NA12878. To evaluate the impact of coverage in the post-processing, each full dataset (66-100x) was down-sampled to generate three subsets with 40, 20 and 10x coverage, respectively. To take into account the sequence divergence, we limited the analysis to chromosome 6 and split it into the HLA region (4-Mb, 29,500,000-33,500,000 bp, hg19; 29,532,223-33,532,223 bp, hg38) of high sequence divergence and the flanking non-HLA regions with low sequence divergence.

**Fig. 4:**
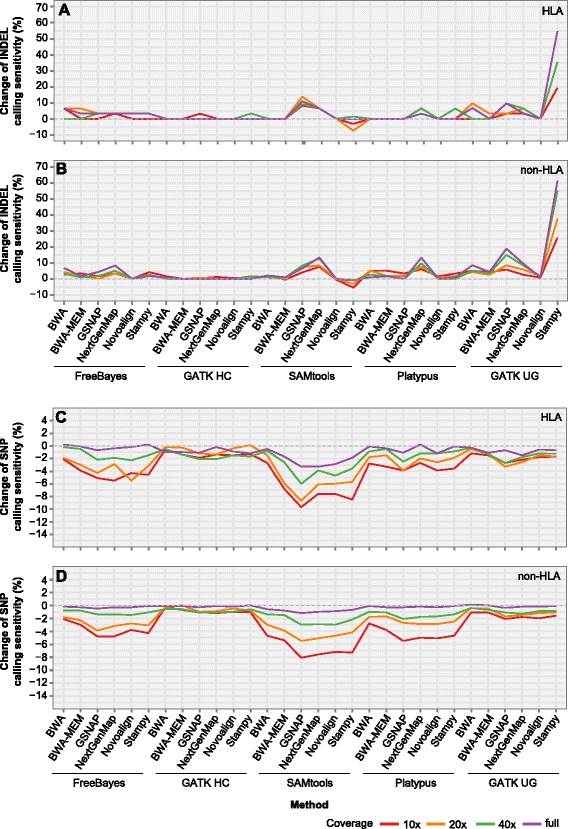
Change of variant calling sensitivity after local realignment and BQSR in NA12878. **a**–**b** Change of INDEL calling sensitivity after local realignment in the HLA (**a**) and non-HLA regions (**b**). **c–d** Change of SNP calling sensitivity after BQSR in the HLA (**c**) and non-HLA regions (**d**). NA12878 exome-seq data NA12878_04 (approximate 100x coverage, Additional file 2: Table S1) was downloaded from https://basespace.illumina.com/and down-sampled into 40, 20 and 10x coverage. Change of sensitivity is calculated as the sensitivity after BQSR, subtracted by that after local realignment, or as the sensitivity after local realignment, subtracted by that after duplicate marking

In SNP calling, the impact of local realignment on sensitivity is only marginal (<=0.5 % change), as observed in the simulated data (Additional file 2: Table S2). In both HLA and non-HLA regions, over 97 % of the cases showed < =0.2 % change in sensitivity. In INDEL calling, the effect of local realignment depends on mapper, caller and coverage. Since the trend is highly comparable across all the five NA12878 datasets, only the results from NA12878_04 are displayed (Fig. 4a and b).

Of the six mappers, Novoalign was impacted the least by local realignment. As for the callers, local realignment had little effect on the sensitivity of GATK HaplotypeCaller in INDEL calling, but a more obvious effect was observed using SAMtools and GATK UnifiedGenotyper, consistent with our observation on simulated data. For these two callers, local realignment generally increases the sensitivity on the alignment from GSNAP and NextGenMap. However, the two callers are less effective in INDEL detection (Tian et al., unpublished results). SAMtools/BCFtools (v1.2) provides two modes of variant detection: consensus-caller described above that assumes only biallelic sites in the genome and multiallelic-caller. We tested local realignment for SAMtools/BCFtools multiallelic-caller. As shown in Additional file 1: Figure S2A, local realignment had similar effects on the two calling modes, with the change of INDEL calling sensitivity being highly correlated (*R*^2^ = 0.941). Further work is needed to test whether the same pattern will be observed in the cancer genome, where more complex structure variation would be generally expected.

INDELs are more difficult to detect and often under-reported [40]. On chromosome 6, the ratio of known INDELs to SNPs is roughly 1–10 in the non-HLA regions and 1–20 in the HLA region based on the public call set [5, 35]. Thus, there are only small changes in the number of INDEL calls, with the exception of Stampy + GATK UnifiedGenotyper. This method showed the most striking increase in sensitivity across the full range of coverage. Thus, the benefit of local realignment in INDEL calling is not universal but mapper- and caller-dependent.

### Impact of BQSR in NA12878 exome data

In SNP calling from simulated data, we observed a general decrease of sensitivity at low coverage and/or high divergence (Fig. 2). Using the NA12878 exome-seq data, we found that BQSR decreased SNP calling sensitivity (i.e., missed known SNPs annotated in the public call set) for majority of the methods, especially at low coverage (Fig. 4c and d; Additional file 1: Figure S3A–H). The pattern in the change of SNP sensitivity across different methods was highly consistent across all five datasets, showing that BQSR had the greatest impact on SAMtools, followed by FreeBayes and Platypus. At full coverage, the effect of BQSR was much reduced in both HLA and non-HLA regions; only SAMtools still showed a noticeable loss of sensitivity in the HLA region. The two SAMtools/BCFtools calling modes showed similar trends in the change of SNP calling sensitivity after BQSR (*R*^2^ = 0.969; Additional file 1: Figure S2B), with multiallelic-caller being slightly less affected than the consensus-caller. Two previous studies also revealed little effect of BQSR in high-coverage whole genome and exome sequencing data [12, 20].

**Fig. 5:**
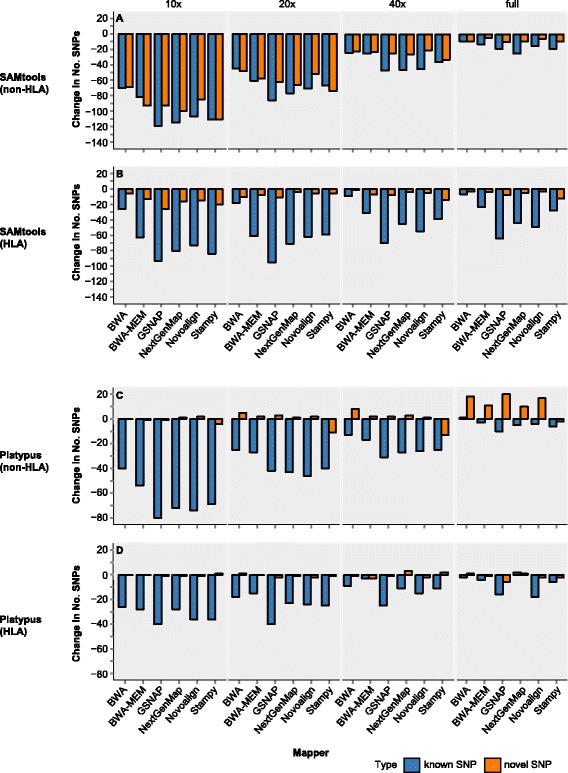
Change of known and novel SNP calls by BQSR in NA12878. **a** SNP calls from SAMtools in the non-HLA regions. **b** SNP calls from SAMtools in the HLA region. **c** SNP calls from Platypus in the non-HLA regions. **d** SNP calls from Platypus in the HLA region. NA12878 exome-seq data NA12878_04 (full, approximate 100x coverage; Additional file 2: Table S1) was down-sampled into 40, 20 and 10x coverage. *Y*-axis represents the difference in the number of SNPs, separated into known and novel, before and after BQSR. Negative number indicates missed calls after BQSR, compared to local realignment. Known SNPs are those that match dbSNP v138

Since BQSR tended to reduce SNP calling sensitivity, we then ask whether loss of sensitivity would be compensated by an increase in precision rate. Precision rate is difficult to estimate for real sequencing data as the false positives are not known. Assuming that some of the novel SNPs represent false positive calls, if BQSR reduces novel SNP calls, it should be an indication of improvement in precision rate. Therefore, we assessed the impact of BQSR on the detection of known versus novel SNPs in all the five datasets. This analysis revealed two general trends, and we illustrated such trends on two of the datasets: NA12878_01 (Additional file 1: Figure S4A–D) and NA12878_04 (Fig. 5a–d).

For SAMtools (Fig. 5a; Additional file 1: Figure S4A) and FreeBayes calling (data not shown) in the non-HLA regions, BQSR resulted in a marked loss of both known and novel SNPs at 10 and 20x coverage, but less so at 40x and full (66-100x) coverage. In the HLA region, however, the loss was much more obvious for known SNPs than for novel ones across all the coverage depths (Fig. 5b; Additional file 1: Figure S4B). Over 90 % (median) of the known SNPs missed by BQSR in the HLA and non-HLA regions overlapped the public call set, supporting the reliability of those missed calls. Thus, in the non-HLA regions, when the coverage is less optimal, BQSR increases the precision rate with sacrifice in sensitivity, indicating a trade-off between these two measurements. However, when the coverage is sufficient, the impact of BQSR becomes much less conspicuous. On the other hand, in the HLA region, BQSR reduces the sensitivity but with little gain in precision rate. On the other hand, for Platypus (Fig. 5c and d; Additional file 1: Figures S4C and D), GATK UnifiedGenotyper and HaplotypeCaller (data not shown), BQSR led to a much larger loss of known SNPs than novel SNPs, particularly at 10 and 20x coverage. For these three callers in SNP detection, the role of BQSR is actually adverse rather than beneficial.

We also investigated the impact of BQSR on INDEL calling. A total of 500 cases were assessed, which represent combinations among 5 NA12878 exome data, 4 coverage depths, 5 mappers (BWA-MEM not included) and 5 callers. In each of the 500 cases, INDELs were first grouped into known (in dbSNP v138) and novel, and both were then split into those that overlap the public call sets and others that are method-specific. For each of the four groups of INDELs in each case, we checked the change in the number of INDELs before and after BQSR (Additional file 1: Figures S5A-D). For FreeBayes, GATK UnifiedGenotyper, Platypus and SAMtools, the number of INDELs differed by no more than two in nearly all the cases in the HLA region and in 95–100 % of the cases in the non-HLA regions. Slightly more changes were observed for GATK HaplotypeCaller in the non-HLA regions, more notably (up to 10 INDELs) for novel ones that are unique (missed in the public call set) to this caller. Overall, BQSR resulted in little or no changes in INDEL calls in both HLA and non-HLA regions.

Taken together, our analysis suggested that the effect of BQSR depends on caller, coverage and sequence divergence. In both high and low divergence regions, BQSR reduced SNP calling sensitivity for all the callers. However, it improved the precision rate only for FreeBayes and SAMtools in the lowly divergent regions when the coverage is insufficient. On the other hand, INDEL calling appears to be less impacted.

### Local realignment and BQSR in hg38 genome reference

Human hg38 reference sequence includes 525 ALT contigs from the HLA region. To evaluate the effect of local realignment and BQSR in this new genome build, we mapped all NA12878_01 and NA12878_04 reads to hg38 using BWA-MEM. We also tested Novoalign (v3.02.04), but it is very slow when ALT contigs are included in the reference (data not shown). The other four aligners are not able to map reads to hg38 if ALT contigs are included. We found that the effect of local realignment on INDEL calling was not markedly different between the two references (Additional file 1: Figure S6A and B). For both references, in the HLA region there was nearly no change (0–1) in the number of known INDELs after local alignment. In the non-HLA regions, local alignment increased the number of known INDELs by 0–5 in hg19 and by 0–3 in hg38, with a difference of < =2 between them. As revealed for hg19, at the full (100x) coverage BQSR mainly reduced the SNP sensitivity of SAMtools when reference hg38 was used (Additional file 1: Figure S6C and D); the effect was more obvious in the HLA region.

## Conclusions

Post-alignment processing is frequently applied in current protocol of variant discovery. Using exome data we revealed that local realignment and BQSR did not always enhance variant detection as one would expect. Instead, their roles are mapper and caller dependent, often varying with coverage depth and level of divergence.

Local realignment and BQSR mainly impacted INDEL and SNP calling, respectively. Local realignment overall increased INDEL calling sensitivity with NextGenMap alignment but had little impact on Novoalign. On the other hand, compared with the haplotype-based callers, the effect was more obvious on SAMtools and GATK UnifiedGenotyper that are less effective in INDEL detection. For majority of the methods, BQSR reduced the SNP calling sensitivity, more obvious at lower coverage. In the low divergence regions, when the coverage is not sufficient, SAMtools and FreeBayes showed decrease in sensitivity but increase in precision rate by BQSR. In other cases, the loss of sensitivity was not associated with an increase in precision rate, which argues against the application of BQSR in those instances. Our analysis offers a broad view about the impact of post-alignment processing in exome-based variant discovery. Thus, consideration should be given to both mapper and caller when deciding whether to apply post-processing to Illumina exome data.

## Additional files

Additional file 1: Figure S1. Change of INDEL calling precision rate following local realignment. **Figure S2:** Local realignment and BQSR for SAMtools/BCFtools consensus-caller and multiallelic-caller. **Figure S3:** BQSR changed variant calling sensitivity in NA12878. **Figure S4:** Change of known and novel SNPs in NA12878 after BQSR. **Figure S5:** Change of known and novel INDELs in NA12878 by BQSR. **Figure S6:** Local realignment and BQSR with hg19 versus hg38. (PDF 769 kb) Additional file 2: Table S1. Five public exome-seq data in NA12878. **Table S2:** Change of SNP calling sensitivity and precision rate after local realignment. (PDF 49 kb)

## Abbreviations

Bp: Base pair
BQSR: Base quality score recalibration
GATK: Genome analysis toolkit
HLA: Human leukocyte antigen
INDEL: Insertion and deletion
PCR: Polymerase chain reaction
SNP: Single nucleotide polymorphism
