# Supplementary material for: Impact of post-alignment processing in variant discovery from whole exome data
Source: BMC Bioinformatics. 2016 Oct 3;17:403. doi: 10.1186/s12859-016-1279-z (PMC5048557; doi:10.1186/s12859-016-1279-z)
Supplement: Additional file 1: Figure S1. — Change of INDEL calling precision rate following local realignment. Figure S2: Local realignment and BQSR for SAMtools/BCFtools consensus-caller and multiallelic-caller. Figure S3: BQSR changed variant calling sensitivity in NA12878. Figure S4: Change of known and novel SNPs in NA12878 after BQSR. Figure S5: Change of known and novel INDELs in NA12878 by BQSR. Figure S6: Local realignment and BQSR with hg19 versus hg38. (PDF 769 kb) [file 12859_2016_1279_MOESM1_ESM.pdf]

Additional file 1

Supplementary methods

1. Parameter settings for mappers

BWA-backtrack (referred to as BWA, v0.5.9) [1] was run with the following parameters:

bwa aln ref.fa end1.fastq > end1.sai
bwa aln ref.fa end2.fastq > end2.sai
bwa sampe -a 1000 -f out.sam ref.fa end1.sai end2.sai end1.fastq end2.fastq
[2]

BWA-MEM (v0.7.12) [3] was run using the command:
bwa mem -M ref.fa end1.fastq end2.fastq > out.sam

GSNAP (v2013-10-25) [4] was run with the following options:
gsnap -d ref.gmapdb -D ref.gmapdb -k 13 --orientation FR --max-mismatches 0.1 --maxsearch 1 --npaths 1 --ordered --show-refdiff -A sam end1.fastq end2.fastq > out.sam

NextGenMap (v0.4.9) [5] was run with the following options:
ngm -r ref.fa -1 end1.fastq -2 end2.fastq -k 15 -I 0 -X 800 -i 0.8 -n 1 -p -o out.sam

Novoalign (v3.01.011, www.novocraft.com) was run with the following options:
novoalign --hdrhd off -i PE 425,80 -r Random -F STDFQ -v 90 -x 5 -o SAM -d ref.nix -f end1.fastq end2.fastq > out.sam

Stampy (v1.0.21) [6] was run with the following options:
stampy.py -g ref -h ref --substitutionrate=0.1 -f sam --inputformat=fastq -o out.sam -M end1.fastq end2.fastq (for simulated data)

stampy.py -g ref -h ref --substitutionrate=0.1 -f sam --inputformat=fastq --xa-max=3 --xa-max-discordant=10 -o out.sam -M end1.fastq end2.fastq (for real data)

2. Parameter settings for callers

GATK UnifiedGenotyper (v2.7-2) [7, 8]:

java –jar GenomeAnalysisTK.jar -T UnifiedGenotyper -R ref.fa -L chr6 -L chr6.interval_list -isr intersection -glm BOTH -mbq 17 --dbsnp dbsnp_138.hg19.vcf.gz -stand_call_conf 20 -stand_emit_conf 10 -o out.vcf -I align.bam

Note: chr6.interval_list is in the format of chr:start-end.

GATK HaplotypeCaller (v2.7-2) [7, 8]:

java –jar GenomeAnalysisTK.jar -T HaplotypeCaller -R ref.fa -L chr6 -L chr6.interval_list -isr intersection --genotyping_mode DISCOVERY --dbsnp dbsnp_138.hg19.vcf.gz -stand_call_conf 20 -stand_emit_conf 10 -o out.vcf -I align.bam

FreeBayes (v9.9.2-27) [9]:

freebayes -f ref.fa -t chr6.bed -C 2 -3 40 -P 0.0001 -m 0 -q 17 -W 1,3 -S 4 -M 3 -B 25 -E 3 -v out.vcf -b in.bam

Note: chr6.bed is in the format of chr<TAB>start<TAB>end

Platypus (v0.5.2) [10]:

Platypus.py callVariants --refFile=ref.fa --regions=chr6.interval_list --ploidy=2 --maxVariants=15 --minBaseQual=17 --output=out.vcf --bamFiles=align.bam --maxVariants=50 minMapQual=0 --rmsmqThreshold=0 --hapScoreThreshold=0

SAMtools mpileup (v0.1.19) and BCFtools (consensus-caller) [11]:

samtools mpileup -ugf ref.fa -l chr6.bed -q 0 -Q 17 -B -F 0.002 align.bam | bcftools view -bvcg - > out.bcf

bcftools view out.bcf > out.vcf

SAMtools mpileup (v1.2) and BCFtools multiallelic-caller [11]:

samtools mpileup -ugf ref.fa -l chr6.bed -q 0 -Q 17 -B -t DP -F 0.002 align.bam |

bcftools call -vmO z - > temp.out.vcf.gz

bcftools norm -m -both -O v -f temp.out.vcf.gz > out.vcf

References

1. Li H, Durbin R. Fast and accurate short read alignment with Burrows-Wheeler transform. Bioinformatics. 2009;25(14):1754-60.
2. Zhao H, Sun Z, Wang J, Huang H, Kocher JP, Wang L. CrossMap: a versatile tool for coordinate conversion between genome assemblies. Bioinformatics. 2014;30(7):1006-7.
3. Li H. Aligning sequence reads, clone sequences and assembly contigs with BWA-MEM. arXiv:13033997. 2013.
4. Wu TD, Nacu S. Fast and SNP-tolerant detection of complex variants and splicing in short reads. Bioinformatics. 2010;26(7):873-81.
5. Sedlazeck FJ, Rescheneder P, von Haeseler A. NextGenMap: fast and accurate read mapping in highly polymorphic genomes. Bioinformatics. 2013;29(21):2790-1.
6. Lunter G, Goodson M. Stampy: a statistical algorithm for sensitive and fast mapping of Illumina sequence reads. Genome Res. 2011;21(6):936-9.
7. McKenna A, Hanna M, Banks E, Sivachenko A, Cibulskis K, Kernytsky A, Garimella K, Altshuler D, Gabriel S, Daly M et al. The Genome Analysis Toolkit: a MapReduce framework for analyzing next-generation DNA sequencing data. Genome Res. 2010;20(9):1297-303.
8. DePristo MA, Banks E, Poplin R, Garimella KV, Maguire JR, Hartl C, Philippakis AA, del Angel G, Rivas MA, Hanna M et al. A framework for variation discovery and genotyping using next-generation DNA sequencing data. Nat Genet. 2011;43(5):491-8.
9. Garrison E, Marth G. Haplotype-based variant detection from short-read sequencing. http://arxiv.org/abs/1207.3907v2. 2012.
10. Rimmer A, Phan H, Mathieson I, Iqbal Z, Twigg SR, Wilkie AO, McVean G, Lunter G. Integrating mapping-, assembly- and haplotype-based approaches for calling variants in clinical sequencing applications. Nat Genet. 2014;46(8):912-8.
11. Li H, Handsaker B, Wysoker A, Fennell T, Ruan J, Homer N, Marth G, Abecasis G, Durbin R. The Sequence Alignment/Map format and SAMtools. Bioinformatics. 2009;25(16):2078-9.

Supplementary Figures

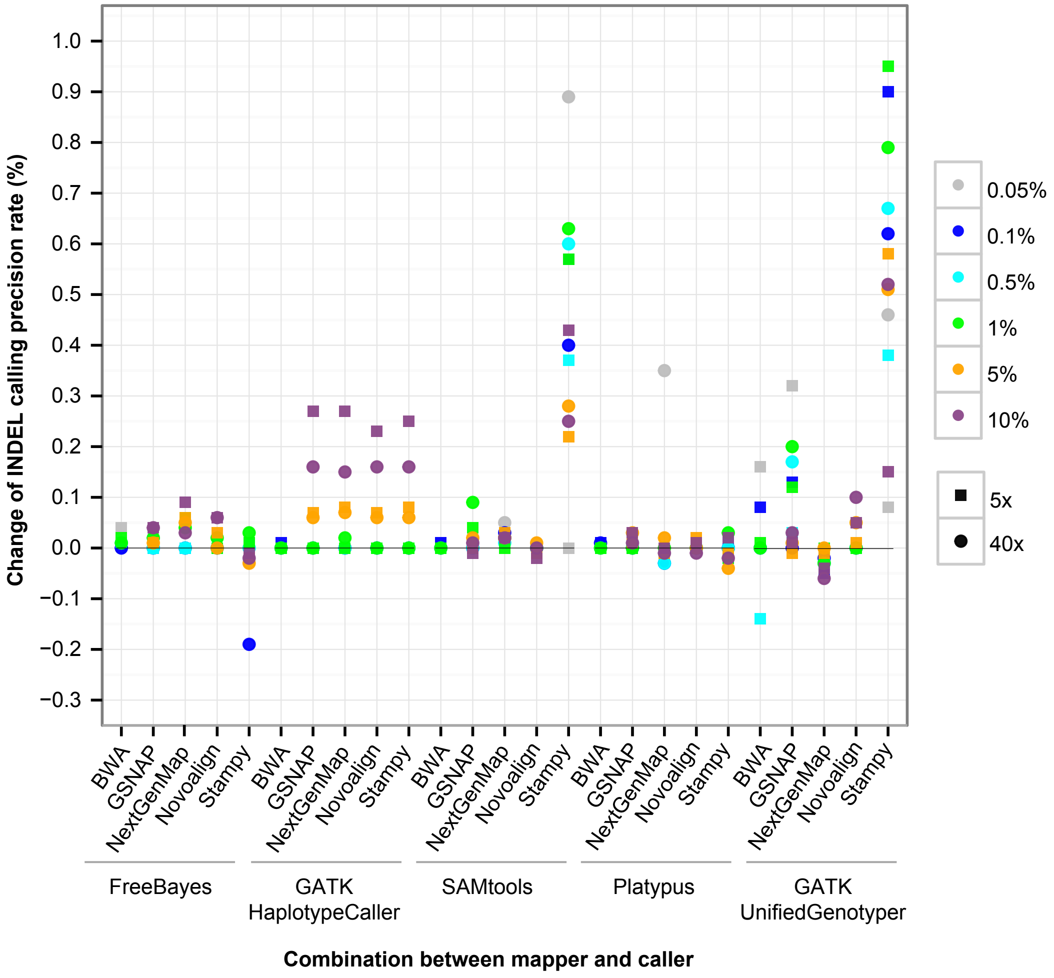

Figure S1 Change of INDEL cFailglinugrepSre2cision rate following local realignment. The change in precision rate is calculated as the precision rate after local realignment, subtracted by that after duplicate marking. For each of the mapper- caller combinations (x-axis), the plot shows the distribution of changes in precision rate across six divergence levels (0.05-10%, by color) and two coverage depths (5x and 40x, by shape). For the combinations with BWA mapping, only 0.05-1% divergence levels are shown.

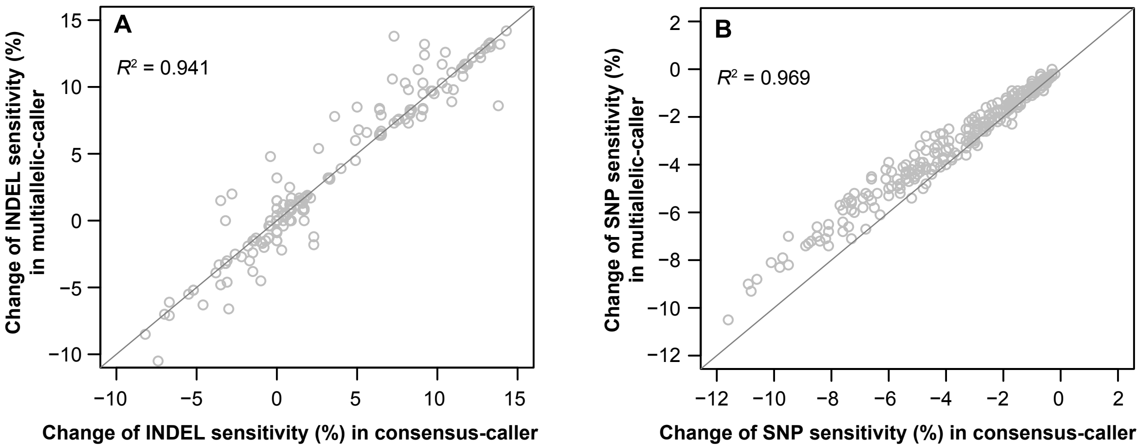

Figure S2 Local realignment and BQSR for SAMtools/BCFtools consensus-caller and multiallelic-caller. (**A**) Scatter plot of the change in INDEL calling sensitivity after local alignment. (**B**) Scatter plot of the change in SNP calling sensitivity after BQSR. Reads from five NA12878 exome data (Additional file 2: Table S1) at 10x, 20x, 40x and full coverage were mapped to the hg19 reference sequence using six mappers. Alignments were subjected to duplicate marking, local realignment and BQSR. For each of the 120 datasets (5 exomes, 4 coverage depths and 6 mappers), variants were identified by SAMtools/BCFtools consensus-caller and multiallelic-caller. Change of known INDEL calling sensitivity (**A**) was estimated from the sensitivity of local realignment, subtracted by that of duplicate marking. Change of known SNP calling sensitivity (**B**) was estimated from the sensitivity of BQSR, subtracted by that of local realignment. The coefficient of determination (R^2) was calculated using Pearson correlation coefficient.

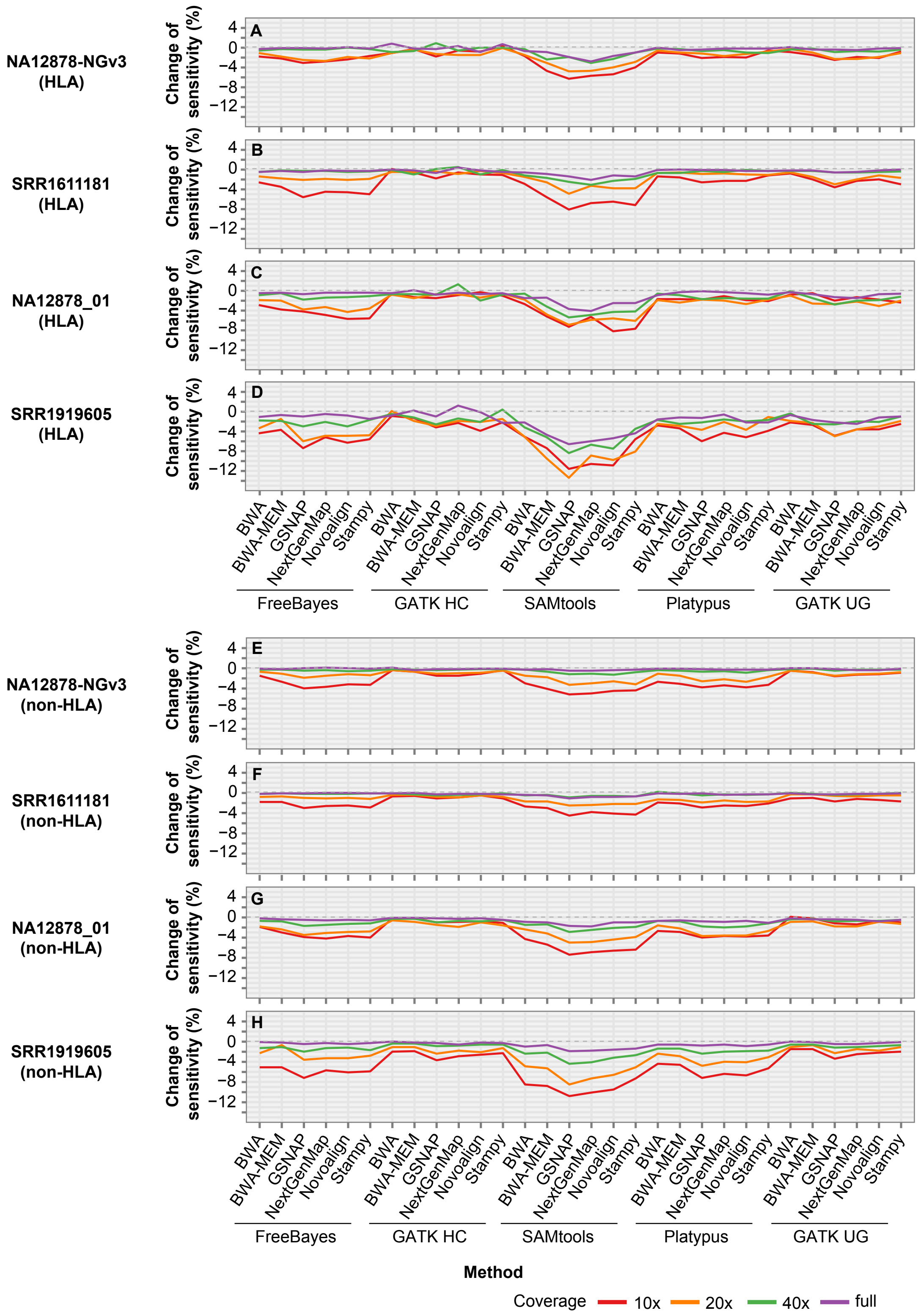

Figure S3 BQSR changed SNP calling sensitivity in four NA12878 datasets. See Figure 4 legend for details.

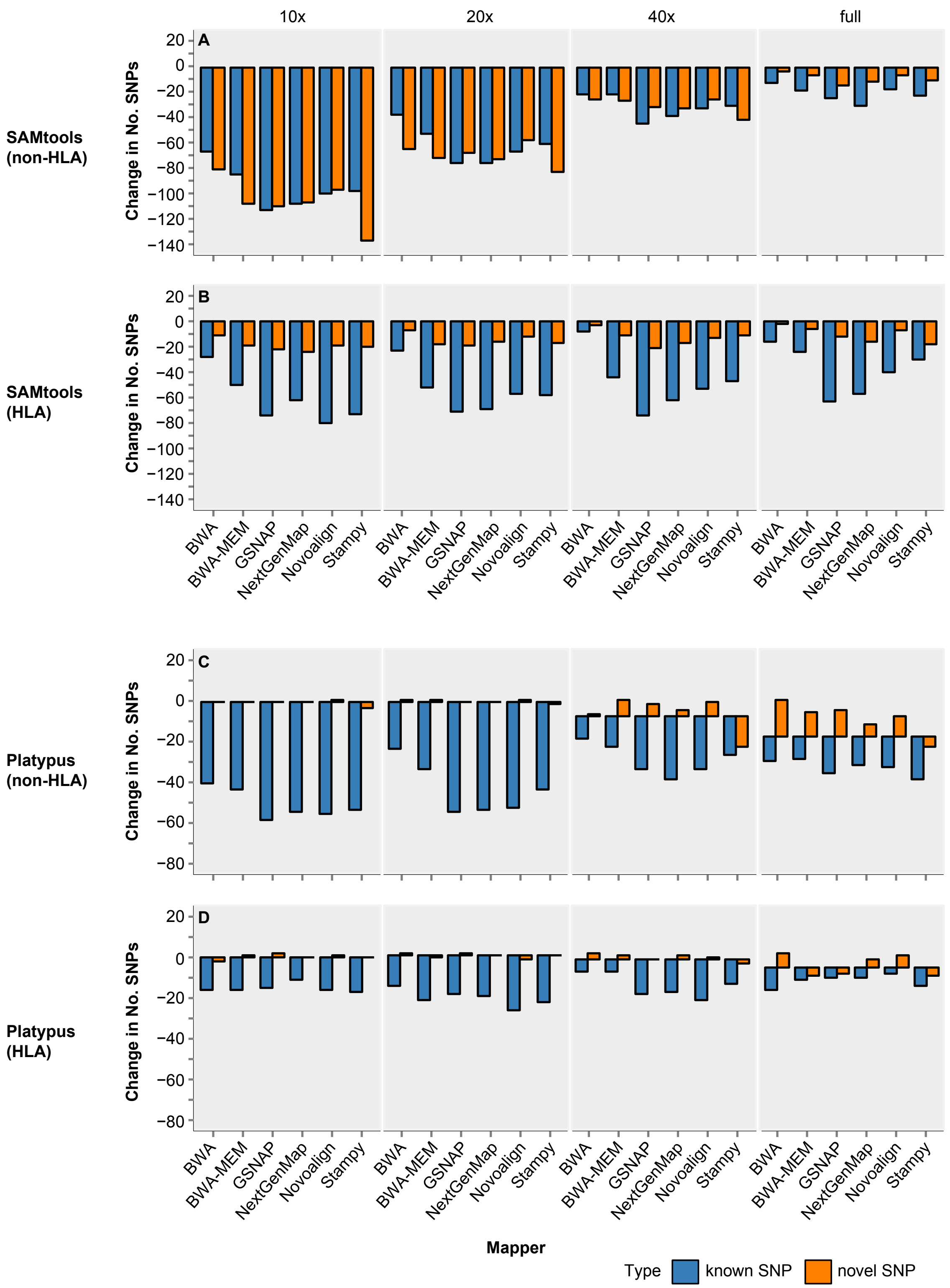

Figure S4 Change of known and novel SNPs in NA12878 after BQSR. SNPs were called from exome-seq data NA12878_01 and separated into known and novel. See Figure 5 legend for details.

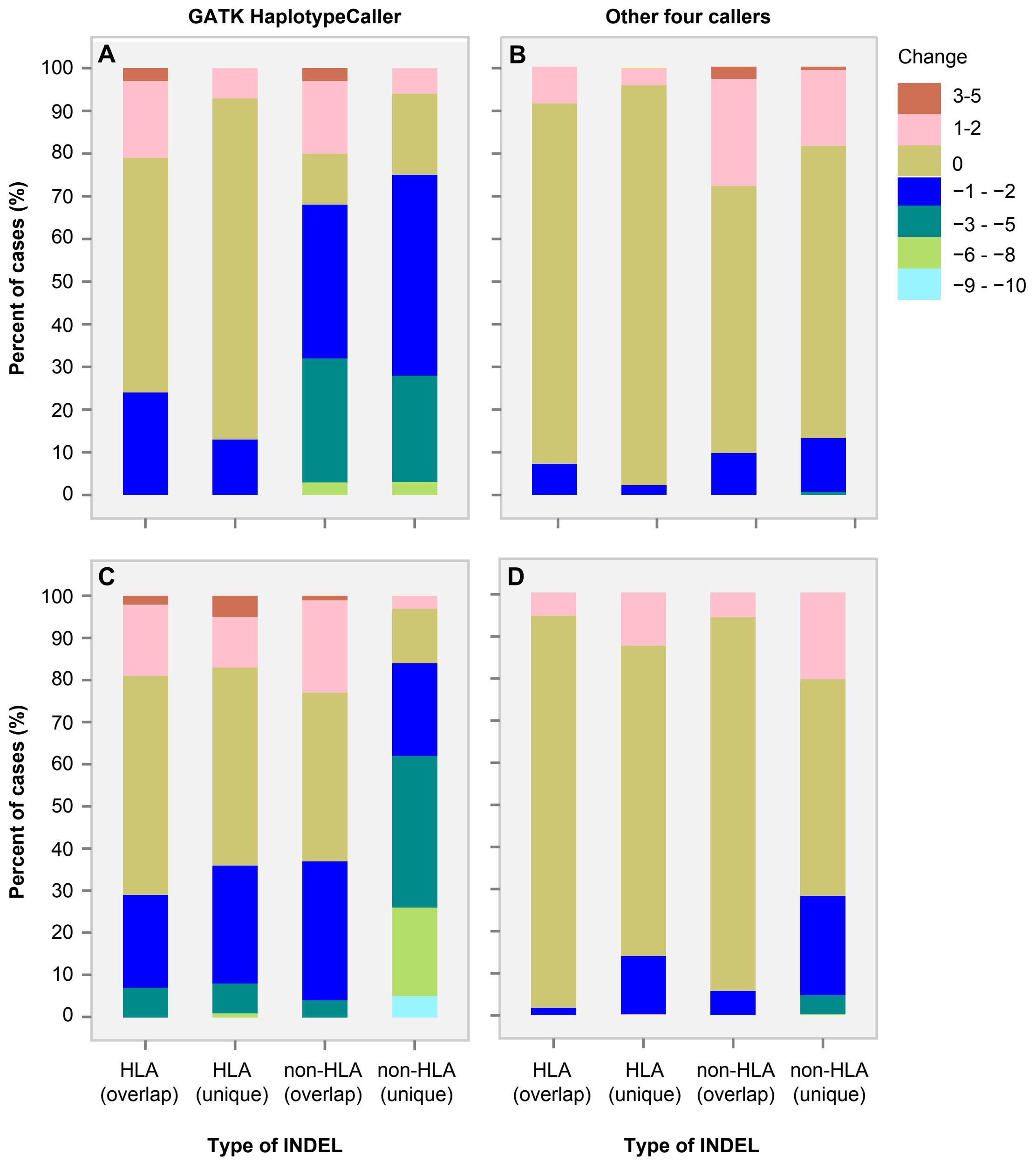

Figure S5 Change of known and novel INDELs in NA12878 by BQSR. INDELs were called from five NA12878 exome-seq data both before and after BQSR. For each data, the full coverage dataset (about 66-100x) and three subsets of 10x, 20x and 40x coverage were used. INDELs were separated into known (**A** and **B**) and novel (**C** and **D**) by intersecting with dbSNP v138, and both were then compared to the public call set to identify overlapped and method-specific calls.

A total of 500 cases, with 100 cases from GATK HaplotypeCaller and 400 cases from the other four callers, were assessed. These 500 cases were from five exome-seq data, four coverage depths, five mappers (BWA-MEM not included) and five callers. Each case was assessed for change in the number of known and novel INDELs after BQSR, with positive number indicating increase and negative number indicating decrease of INDELs. Y-axis represents the proportion of cases with varying number of change in INDELs.

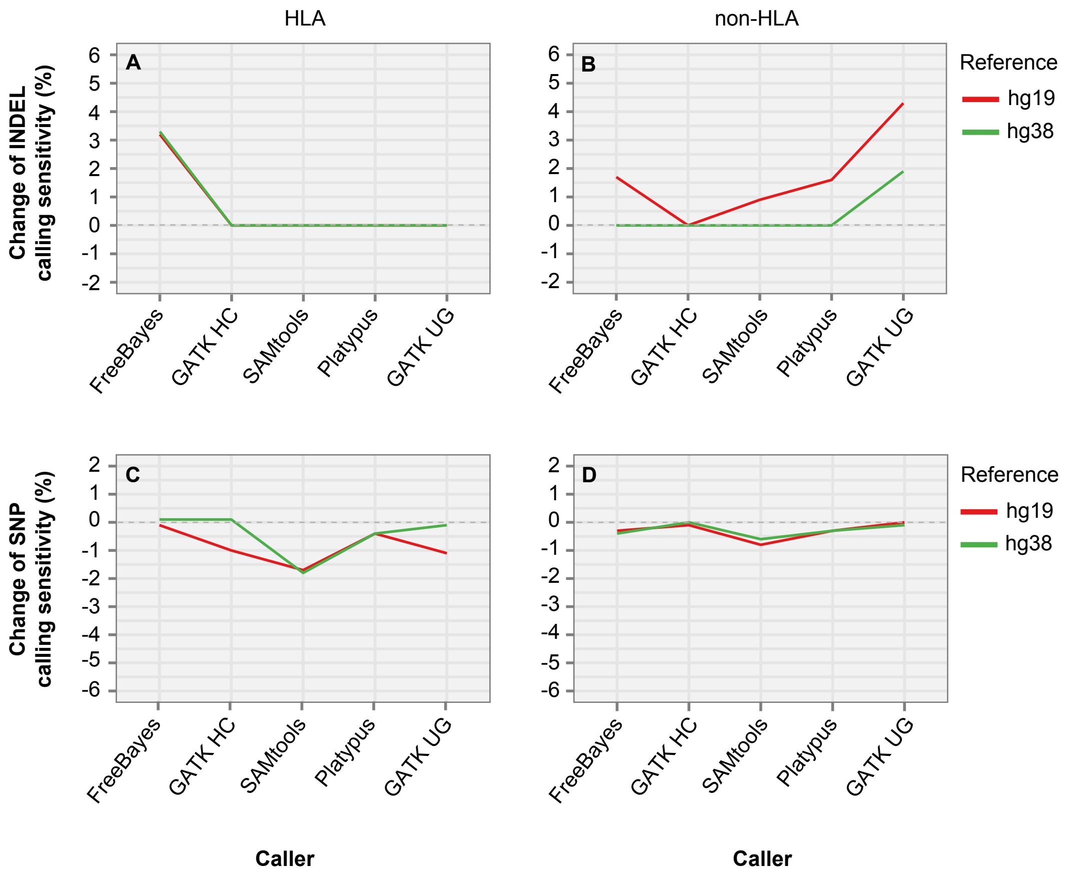

Figure S6 Local realignment and BQSR with hg19 versus hg38. (**A-B**) Change of INDEL calling sensitivity after local alignment. (**C-D**) Change of SNP calling sensitivity after BQSR. Reads from NA12878_04 (Additional file 2: Table S1) at full coverage were mapped to hg19 and hg38 reference sequences using BWA-MEM mappers. Variants were identified using five callers (x-axis). See Figure 4 legend for more information.
