# Supplementary material for: Impact of post-alignment processing in variant discovery from whole exome data
Source: BMC Bioinformatics. 2016 Oct 3;17:403. doi: 10.1186/s12859-016-1279-z (PMC5048557; doi:10.1186/s12859-016-1279-z)
Supplement: Additional file 2: Table S1. — Five public exome-seq data in NA12878. Table S2: Change of SNP calling sensitivity and precision rate after local realignment. (PDF 49 kb) [file 12859_2016_1279_MOESM2_ESM.pdf]

Additional file 2

Table S1 Five public exome-seq data in NA12878

Sequence ID	Name	Approx. coverage	Length (bp)	Platform	Capture kit
NA12878-NGv3-LAB1360-A	NA12878-NGv3	66	100	HiSeq 2000	Roche
SRR1611181	SRR1611181	90	100	HiSeq 2000	Roche
FC1_NA12878_01	NA12878_01	100	150	HiSeq 2500	Illumina
FC1_NA12878_04	NA12878_04	100	150	HiSeq 2500	Illumina
SRR1919605	SRR1919605	100	76	HiSeq 4000	Illumina

The 76-bp and 150-bp reads are available at https://basespace.illumina.com/. Capture kit: Roche, SeqCap EZ Human Exome kit v3.0 (with 64 Mb targets); Illumina, Nextera Rapid Capture Exome kit (with 37 Mb targets).

Table S2 Change of SNP calling sensitivity and precision rate after local realignment

Metrics	Change (%)	No. cases	% cases
Sensitivity	-0.1	4	1.43
Sensitivity	0	242	86.43
Sensitivity	0.1	33	11.79
Sensitivity	0.2	1	0.36
Precision rate	-0.1	1	0.36
Precision rate	0	224	80
Precision rate	0.1	44	15.71
Precision rate	0.2	10	3.57
Precision rate	0.3	1	0.36

See Table 1 for details.
